# Effect of early initiation of steroid-sparing drugs in patients with bullous pemphigoid

**DOI:** 10.3389/fimmu.2023.1176284

**Published:** 2023-07-06

**Authors:** Inger Johanne Fenne, Guro Askildsen Oftebro, Christian Vestergaard, Anne Sofie Frølunde, Rikke Bech

**Affiliations:** ^1^ Study of Medicine, Faculty of Health, Aarhus University, Aarhus, Denmark; ^2^ Department of Dermato-Venereology, Aarhus University Hospital, Aarhus, Denmark

**Keywords:** bullous pemphigoid, steroid-sparing treatment, immunomodulatory treatment, glucocorticoids, relapse, mortality, cardiac disease

## Abstract

**Introduction:**

Bullous pemphigoid (BP) can be treated using systemic and topical glucocorticoids and/or other immunomodulatory agents. However, the long-term use of systemic glucocorticoids causes severe adverse side effects. This study was aimed at investigating whether the early initiation of corticosteroid-sparing therapy (CST) in BP patients results in better outcomes than late or no CST.

**Method:**

We retrospectively identified all BP patients referred to the tertiary center, of the Department of Dermatology and Venerology, Aarhus University Hospital, Denmark, from 2015 to 2021. Patients’ demographics, comorbidities, treatment, remission of BP, length of admission, relapse, and 1-year mortality were recorded. All patients who received CST were dichotomised into two groups: initiated with CST <28 or >28 days. The groups were compared using t-tests. Additionally, all patients who received CST were compared with those who received systemic glucocorticoids alone. Our cohort was compared with that of a previous study (2006–2013) performed in our department. In 2015, we revised our BP treatment guidelines to include the early initiation of CST.

**Results:**

On comparing the group of patients initiated with CST <28 versus >28 days, we found no significant differences in the complications or mortality between the groups (*p* = 0.63 and *p*=0.79, respectively). The <28 days group had a lower rate of relapse (*p* < 0.05). On comparing data from this study with those from the previous study, conducted before we revised our treatment guideline, we found a reduced initial dose of prednisolone and reduced admission time in this study. No significant differences were found between patients treated with CST and those treated with systemic glucocorticoids alone.

**Conclusion:**

The rate of complications and 1-year mortality did not differ significantly between the two subgroups in this study. The relapse rate was lower in the CST <28 days group than in the CST >28 days group. The initial dose of prednisolone and admission time were reduced in this study compared with those in the previous study performed before the implementation of a local treatment guideline recommending the early initiation of CST.

## 1 Introduction

**Figure 1:**
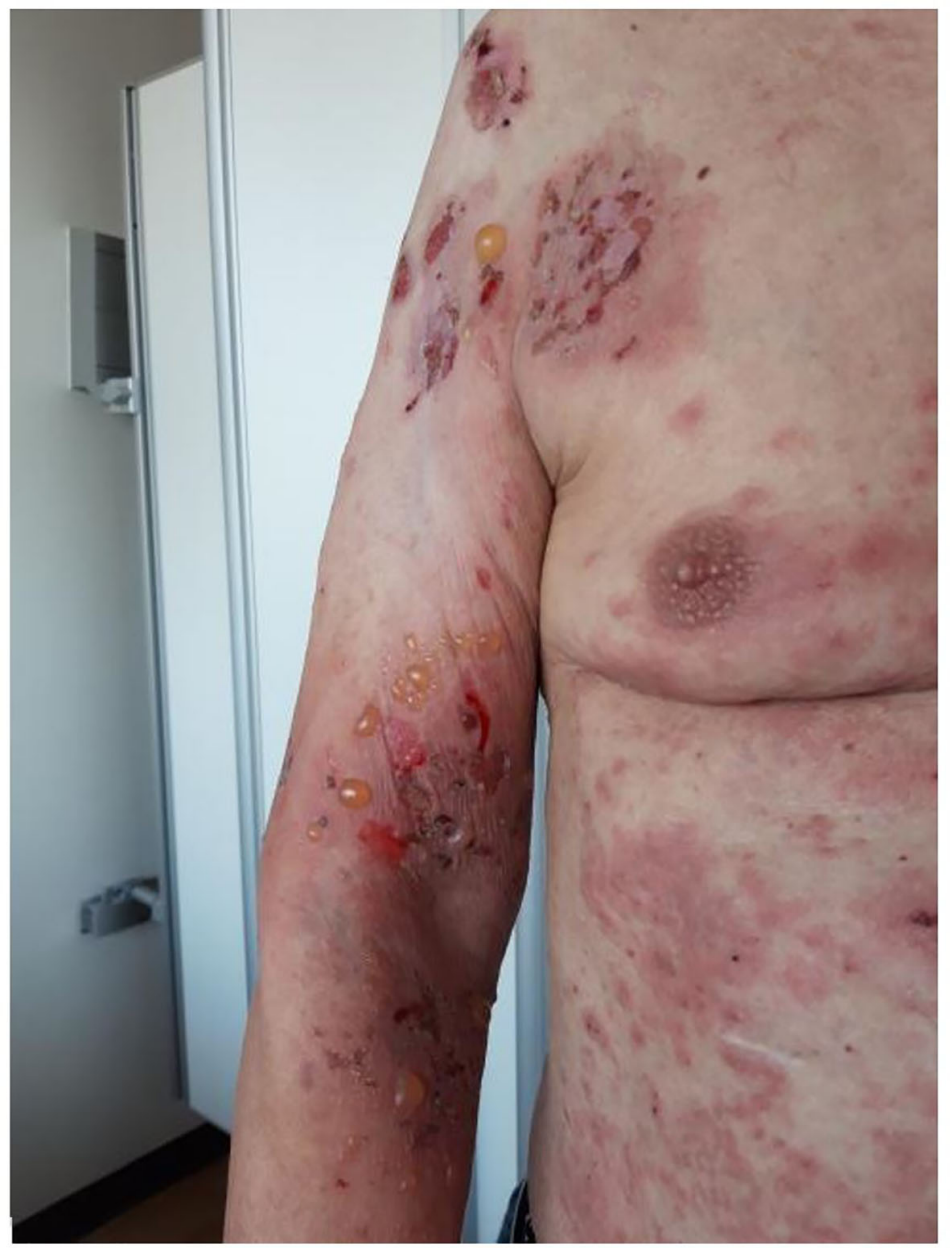
Admitted BP patient with characteristic tense subepithelial blisters and pruritic urticarial plaques.

Bullous pemphigoid (BP) is an autoimmune blistering disease that predominantly affects individuals aged >65 years^1^ and is characterized by tense subepithelial blisters and generalized pruritic urticarial plaques arising from normal or erythematosus skin (1) (**Figure 1**). In one-third of BP patients, the oral and/or anogenital mucosa is also affected. However, one-fifth of patients do not develop blisters; in such patients, the diagnosis is made by direct immunofluorescence of skin biopsy staining for deposits of IgG at the dermo-epidermal border1. Standard therapies include topical and systemic corticosteroids and other immunomodulatory agents. The outcome and prognosis of the disease are correlated with the disease severity. Long-term monitoring is often required, as well as hospitalization in severe cases.

BP is caused by autoantibodies targeting the hemidesmosomal proteins, BP230 (BPAG1) and BP180 (also termed BPAG2 and type XVII collagen). These proteins are components of the dermo-epidermal junction and are important components of the adhesion complexes that promote epithelial-stromal adhesion (2). The binding of antibodies to antigens promotes inflammation by activating the complement system and mast cells. Activated inflammatory cells produce proteolytic enzymes that damage the dermo-epidermal junction. Consequently, subepithelial blisters develop.

The prodromal phase of BP is characterized by pruritus and/or urticarial lesions and detection of circulating autoantibodies in the blood1. As the disease progresses, bullae may develop, which are often located on the flexor surface of the trunk, abdomen, forearms, and medial thighs and in the axillae. The fluid in the bullae is clear, and the bullae can persist for days before leaving erosions and crusts that heal without scarring. Unlike other blistering diseases, such as pemphigus vulgaris, the Nikolsky sign is negative in typical cases of BP whereas the Asboe–Hansen sign is positive (3). Damaged skin can lead to secondary *Staphylococcus* or *Streptococcus* infections, which can be fatal in elderly patients, in case of sepsis1.

The first-line treatment for BP is systemic corticosteroids; however, the choice of treatment also depends on the disease severity and patients’ comorbidities. In mild cases, potent topical steroids are sufficient, whereas in moderate-to-severe disease, systemic immunosuppressive treatments may be needed, including systemic corticosteroids at dosages of up to 0.5 to 0.75 mg/kg/day for the severe form of BP. This consensus on BP treatment with lower doses of systemic corticosteroids was developed by an international panel of experts in the field of autoimmune blistering disorders (4). Higher doses of glucocorticoids may be more effective in disease control but are not advisable because of the associated risk of adverse effects and increased 1-year mortality (5, 6).

A Finnish study from 2016 indicated that an early initiation of corticosteroid-sparing therapy (CST) and limitation of the systemic steroid dose to a maximum of 0.75 mg/kg/day may increase the survival of BP patients (7). The local guidelines at the Department of Dermatology at Aarhus University Hospital were revised in 2015 to recommend the systematic introduction of CST to BP patients within the first day of admission, in addition to the regular dose of prednisolone 0.5–0.75 mg/kg/day. If the patient did not tolerate prednisolone, tetracycline was administered. Azathioprine 0.5–2.5 mg/kg/day is often used as a steroid-sparing drug. Alternatively, other steroid-sparing drugs such as methotrexate, mycophenolate mofetil, or cyclosporine may be used depending on the individual patient and discretion of the treating doctor. In treatment-refractory cases, intravenous immunoglobulins, plasmapheresis, or rituximab may be administered.

To differentiate between different clinical severities of BP, two scoring systems have been developed: the Autoimmune Bullous Skin Disorder Intensity Score (ABSIS) and the Bullous Pemphigoid Disease Area Index (BPDAI) (8, 9). The BPDAI was developed by the International Pemphigoid Committee and is based on three subcomponents: cutaneous blisters, erythema/urticaria, and mucosal blisters/erosions (8). ABSIS uses a combination of the percentage of body surface involved and a subsequent weighting factor based on whether a lesion is exudative, dry, or re-epithelialized (9). However, these two scoring systems are not always employed in clinics as they are rather comprehensive and, therefore, more suitable for research purposes.

This study aimed to retrospectively describe the demographics, comorbidities, treatment (pre-hospitalization medications; glucocorticoids, including doses; and steroid-sparing drugs), and treatment outcomes (remission, length of admission, relapse, and 1-year mortality) in a cohort of all BP patients referred to a tertiary centre (Department of Dermatology, Aarhus University Hospital) from 2015 to 2021. We hypothesized that consistent application of the new local guidelines (early initiation of CST) in the department would improve the survival rate, lower the rate of complications, and lower the rate of relapse, thereby lowering the number of readmission and length of admission. In this manner, the new guidelines can be evaluated to provide valuable information about the effects of current steroid-sparing treatments. Our secondary objective was to compare our findings regarding comorbidities and pre-hospitalization medications with existing literature to contribute to the understanding of possible associations and epidemiology of BP.

## 2 Materials and methods

### 2.1 Design and patients

**Figure 2:**
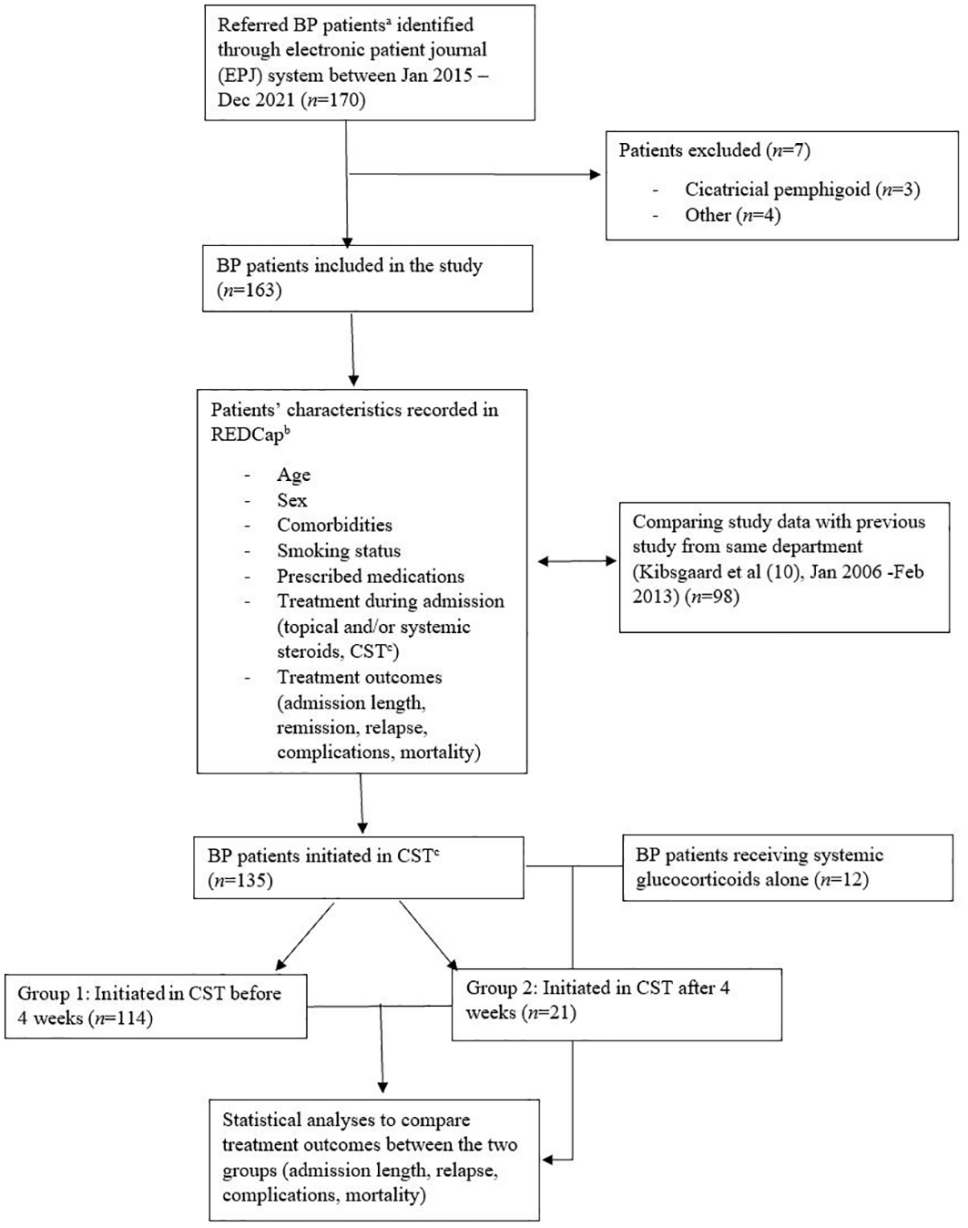
Flow chart of study design and patients. ^a^Referred to the Department of Dermatology and Venerology at Aarhus University Hospital; ^b^The Research Electronic Data Capture; ^c^Corticosteroid-sparing therapy (CST).

In this retrospective case-series study, the medical records of all referred patients diagnosed with BP at the Department of Dermatology and Venerology, Aarhus University Hospital, from 2015 to 2021 were investigated (**Figure 2**). The cohort was a heterogeneous group of patients, some referred from general practitioners as having suspected BP and some from private dermatology practitioners. Patients were identified using the ICD10 system. The diagnoses included in this study were BP (DL 12.0), unspecified pemphigoid (DL 12.9), and other pemphigoids (DL 12.8). The project was reported to the Regional (Region Midtjylland) Record of Research Projects (registration number: 1-16-02-44-22).

Patient characteristics (age, comorbidities, smoking, and prescribed medicines), treatment during admission, and treatment outcomes (admission length, remission, relapse, and mortality) were recorded using the Research Electronic Data Capture (REDCap) data management platform. Patients were included if characteristic clinical presentations such as bullae and pruritus were found; skin biopsies revealed subepidermal clusters of eosinophils and/or bullae; and/or direct immunofluorescence (IF) showed linear deposits of IgG, IgA, or complement C3 along the basement membrane zone. Patients with primary mucosal involvement were excluded, as this suggested a cicatricial pemphigoid.

The treatment of the referred patients was based on the local guidelines of the Department of Dermatology at Aarhus University Hospital. First-line therapies included potent topical glucocorticoids in combination with prednisolone if needed. Prednisolone was usually administered as 0.5–0.75 mg/kg/day and updosed until effect (no appearance of new blisters). If the patient did not tolerate systemic corticosteroids, tetracycline was administered. Local treatment guidelines recommend the initiation of CST on the day of admission. According to these guidelines, CST can include azathioprine, methotrexate, mycophenolate mofetil, or cyclosporine. In treatment-refractory cases, intravenous immunoglobulins, plasmapheresis, or rituximab can be administered.

### 2.2 Variables

To assess the outcomes, multiple data from medical records were included in our study. Data such as treatment and dosage, length of admission, readmission, relapse, remission, complications after diagnosis (sepsis and/or diabetes), and 1-year mortality were included. The length of admission was used as a proxy for the disease course, as patients were normally discharged when new bullae did not appear for a minimum of 2 days and primary skin lesions started to re-epithelialize. Frequent clinical control following a fixed scheme after discharge was common practice, and in this manner, relapse or remission was recorded. Remission was defined as the absence of bullae and/or urticaria. Readmission within the first year of diagnosis was interpreted as relapse of BP.

### 2.3 Data analysis

We used means and standard deviations (SDs) for all continuous variables and percentages for all categorical variables. To test our hypothesis that the early initiation of CST is related to treatment outcomes, all patients that received CST were secondarily dichotomised into two groups. The first group included patients receiving CST within 4 weeks (<28 days), and the second group included patients who received CST after 4 weeks (>28 days). We investigated the association between earlier initiation of CST and treatment outcomes versus late initiation. Furthermore, we compared all patients receiving CST with those who received prednisolone alone. The treatment outcomes were defined as the number of days admitted, relapse rate, rate of complications (sepsis and/or diabetes), and 1-year mortality. To compare the groups (CST before versus after 4 weeks groups and CST versus prednisolone alone group), we used *t*-tests, which was a two-sample equal variance (homoscedastic) test based on the assumption that the groups originated from the same population. *p*-values <0.05 were considered statistically significant.

## 3 Results

### 3.1 Patients

Between 01/01/2015 and 31/12/2021, a total of 170 patients were identified. Of these patients, three who were retrospectively diagnosed with cicatricial pemphigoid were excluded, along with four patients who were excluded for other reasons. Finally, 163 patients were included in the study (**Figure 2**).

The age at BP diagnosis ranged from 27 to 100 years with a mean ± SD age of 79 ± 10 years. Of the 163 patients, 94 (58%) were men and 69 (42%) were women (**Table 1**).

**Table 1:** Baseline characteristics of patients admitted with bullous pemphigoid (n = 163) during the study period (Jan 2015–Dec 2021).

Characteristics	Patients
Sex, %	
Male	58
Female	42
Age at onset of BP, years, mean ± SD	79 ± 10
Diagnosis methods, %	
Objective	2
+ Histology	10
+ Immunofluorescence	88
Comorbidities, %	
Cardiovascular disease	46
Type II diabetes mellitus	29
Cancer	25
Neurologic disorders^a^	21
Type I diabetes mellitus	1
Other	26
Pre-hospitalisation medications^b^, %	
Paracetamol	41
Acetylsalicylic acid	23
ACE inhibitors	21
Furosemide	17
Gliptins^c^	12
NSAIDs	4
None of the above	31

a Stroke (n = 24), Alzheimer’s disease (n = 5), Parkinson’s disease (n = 3), and multiple sclerosis (n = 3).

b Not included in the table: none of the patients (n = 0) received erlotinib, etanercept, anti-TNF-alfa, rifampicin, nivolumab/pembrolizumab, sirolimus, tiobutarit, or alogliptin. One patient received everolimus. Five patients did not use any medicines at all.

c A total of 18 patients received vildagliptin, one received linagliptin, and one received sitagliptin.

BP diagnosis was based on clinical, histological, and immunofluorescence in 143 (88%) of the 163 patients.

As for comorbidities, the most common comorbidity was cardiovascular diseases (CVDs), which was present in 75 patients (46%), followed by type II diabetes mellitus (DM), which was present in 48 patients (29%), and neurological disorders, including Alzheimer’s disease, Parkinson’s disease, multiple sclerosis, and stroke, which was present in 35 patients (21%) (**Table 1**).

Before hospitalization, the patients were most commonly treated with paracetamol (41%), acetylsalicylic acid (23%), angiotensin-converting-enzyme (ACE) inhibitors (21%), furosemide (17%), and gliptins (12%). None of the included patients were treated with nivolumab, erlotinib, etanercept, rifampicin, or sirolimus at the time of admission (**Table 1**). These drugs have been associated with the development of BP (2).

### 3.2 Treatment outcomes

Of the 163 patients, 106 (65%) were initiated with systemic glucocorticoids. The initial doses ranged from 5 to 50 mg, with a mean ± SD of 25 ± 11 mg (**Table 2**). Of the remaining 57 patients who were not initiated on systemic glucocorticoids at time of the first visit, 23 (40%) were initiated subsequently. We found that 131 (80%) of the 163 patients were initially treated with topical glucocorticoid group 4; 30 and 20% were treated with group 3 and 2 steroids, respectively. Whether the topical treatment was used only on the lesions or whole body (except face and anogenital region) varied based on clinical severity and doctor’s discretion. In addition to topical steroids, some patients also received systemic steroids and/or CST.

**Table 2:** Comparison of treatment and treatment outcomes in patients with bullous pemphigoid at the Department of Dermatology and Venereology at Aarhus University Hospital in two different time periods.

Jan 2006–Feb 2013 (n = 98)*		Jan 2015–Dec 2021 (n = 163)	
Treatment and outcomes	Patients	Treatment and outcomes	Patients
Length of primary admission, days mean ± SD	14 ± 9	Length of primary admission, days mean ± SD	8 ± 5
Dosage of systemic glucocorticoid at admission, mg, mean ± SD^a^	44 ± 15	Dosage of systemic glucocorticoid at admission, mg, mean ± SD^a^	25 ± 11
Corticosteroid-sparing therapy^b^, patients, n		Corticosteroid-sparing therapy^b^, patients, n	
Azathioprine	80	Azathioprine	55
Methotrexate	11	Doxycykline^c^	37
Mycophenolate mofetil	3	Methotrexate	33
Dapsone	1	Dapsone	8
Rituximab	1	Mycophenolate mofetil	1
		Plasmapheresis	1
		Rituximab	0
		Other treatments	0
6-week remission, %	74	1 year remission, %	93
Relapse^d^, %	34	Relapse^d^, %	38
		Complications^e^, %	13
		1-year mortality, %	17

*Data from Kibsgaard et al. (10).

a2006-2013: Nine patients did not receive systemic glucocorticoid and did not contribute to this value (n=89). 2015-2021: 57 patients did not receive systemic glucocorticoids (n=106); b2006-2013: 12 of 98 patients did not receive CST for >1 week (n=86). 2015-2021: 28 of 163 patients did not receive CST (n=135); cTetracycline indicated when oral glucocorticoid treatment is contraindicated; d2006-2013: At any time of the study period. 2015-2021: 1 year after diagnosis. eDefined as diagnosis of sepsis and/or diabetes 1 year after BP diagnosis.

**Figure 3:**
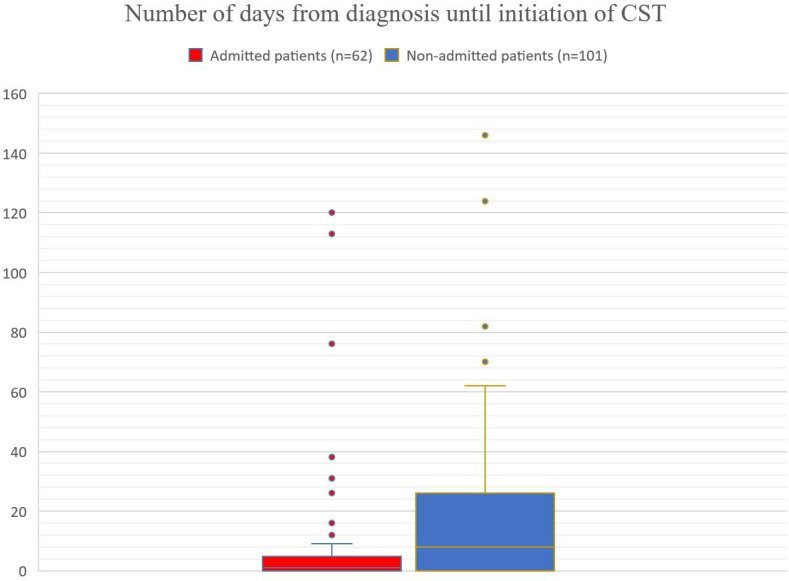
Admitted patients (red): mean of 1 day, number of days ranged from 0 to 120 days, with an upper quartile (75^th^ percentile) of 5 days. Non-admitted patients (blue): mean of 8 days, number of days ranged from 0 to 719 days, with an upper quartile (75^th^ percentile) of 26 days.

Of the 163 patients, 135 (83%) were initiated on CST. The choice of CST was based on an individual assessment by taking into consideration comorbidities (CVD or existing/prior neoplasm), kidney and liver function, age, and performance status. Azathioprine was the most frequently used CST (*n* = 55), followed by doxycycline (*n* = 37) and methotrexate (*n* = 33; **Table 2**). Eight patients received dapsone as adjuvant CST. One patient was treated with mycophenolate mofetil and one with plasmapheresis. None of the patients were treated with rituximab during the study period. The number of days from diagnosis until the initiation of CST ranged from 0 to 719 days, with a mean of 3 days. For admitted patients, the number of days ranged from 0 to 120 days, with a mean of 1 day. For the non-admitted patients, the number of days ranged from 0 to 719 days, with a mean of 8 days (**Figure 3**).

At the time of diagnosis 62 (38%) out of the 163 patients were admitted to the hospital because of the severity of their BP disease. The number of days admitted ranged from 1 to 26, days with a mean ± SD of 8 ± 5 days (**Table 2**). Within the first year after BP diagnosis, 15 (24%) of the 62 admitted patients were readmitted. Of the 163 patients, 151 (93%) were classified as having remission of the disease within the first year and 141 (87%) did not have complications such as sepsis or diabetes after diagnosis. Overall, 17% of the patients died within the first year after diagnosis.

We found that 114 (84%) of those 135 patients initiated on CST were initiated on CST within 28 days (early initiation of CST) and 21 (16%) were initiated after 28 days (late initiation of CST).

In the CST <28 days group, the number of days admitted ranged from 1 to 26 days, with a mean ± SD of 8 ± 5 days, whereas in the CST >28 days group, this number ranged from 1 to 13 days, with a mean ± SD of 8 ± 5 days (*p* = 0.49). The rates of complications (sepsis and/or diabetes 1 year after diagnosis) were 15% and 19% (*p* = 0.63), the relapse rates were 38% and 62% (*p* < 0.05), and the 1-year mortality was 17% and 14% (*p* = 0.79) in the CST <28 days and CST >28 days groups, respectively (**Table 3**).

**Table 3:** Treatment outcomes stratified by the number of days until the initiation of CST.

All patients^a^			
	<28 days (n = 114)	>28 days (n = 21)	*p*-value
Number of days admitted, mean ± SD	8 ± 5	8 ± 5	0.49
Complications^b^, %	15	19	0.63
Relapse, %	38	62	<0.05
1-year mortality, %	17	14	0.79

^a^Of 163 patients, 28 were not initiated on CST (n=135).

^b^Defined as diagnosis of sepsis and/or diabetes 1 year after BP diagnosis. During the study period only one patient was diagnosed with diabetes mellitus.

Only 12 patients (7%) were treated with prednisolone alone, whereas 135 (83%) were treated with CST. Compared with the CST group in which 46% of the patients were admitted to the hospital, none of those treated with prednisolone alone were admitted. The relapse rates were 33% and 42% (*p* = 0.55), the rates of complications were 8% and 16% (*p* = 0.50), and the 1-year mortality was 33% and 16% (*p* = 0.14) in the prednisolone alone and CST groups, respectively. None of the abovementioned data were statistically different between the prednisolone alone and CST groups.

## 4 Discussion

The rate of complications and 1-year mortality did not differ significantly between the groups that initiated early (<28 weeks) or late (>28 weeks) corticosteroid-sparing therapy (CST). Instead, we found a minor increase in 1-year mortality in the <28-days group (17% *vs*. 14%, *p* = 0.79). However, the relapse rate was significantly different between the groups (*p* < 0.05), where those initiated early on CST had fewer relapses. This suggests a positive effect of the early initiation of steroid-sparing drugs. However, the relapse rate may be biased by patient compliance and disease severity; therefore, the outcome must be interpreted with caution.

Considering our cohort and its rather small size (compared with other cohorts of more prevalent diseases), finding significant results can be challenging. By performing a quick power calculation of our two subgroups and outcome relapse, the minimum powerful sample size was 134. As we had a cohort of 135 patients initiated on CST, there were theoretically enough patients to provide our study with statistical power.

There were 163 BP patients referred to the Department of Dermatology and Venerology from 2015 to 2021. This is an increase compared with an almost identical study by Kibsgaard et al. conducted from 2006 to 2013 within the same department (10). Kibsgaard et al. reported that 98 BP patients were referred to the Department of Dermatology during a 7-year study period from 2006 to 2013 (10). The increased number of BP patients could be due to several factors, including a continuously increasing proportion of the elderly population and/or better diagnostics of BP. Additionally, the same trend of an increasing incidence of BP patients has been observed in several other countries the last decades (11, 12). Moreover, other causal factors such as a higher prevalence of neurological diseases have been shown to increase the risk of developing BP (13). The same inclusion criteria were used in Kibsgaard et al.’s study and this study. Therefore, the increased number of referred BP patients may reflect an actual increase in the incidence of BP in the Danish population.

In this study, 38% of the referred patients were admitted to the hospital because of the severity of their BP disease. This was not only due to high disease activity and need for intensive therapy with intravenous treatments in some cases but also because of comorbidities, as all the admitted patients were initiated on CST to reduce the use of systemic steroids.

The time from BP diagnosis to the initiation of CST varied from 0 to 719 days, with a mean of 3 days (**Figure 3**). In the group of admitted patients, the mean was only 1 day, and 75% of the admitted patients were initiated on CST within 5 days. On the other hand, in the group of non-admitted patients, the mean was 8 days, and 75% of the patients were initiated within almost 4 weeks (26 days). Thus, the admitted patients experienced earlier initiation of CST. This supports more strictly application of our treatment guidelines in patients admitted to our inpatient ward compared with those treated in our outpatient clinic. There should be no differences in the handling of admitted/non-admitted patients.

We found no difference in treatment outcomes when comparing the group of BP patients treated with prednisolone alone and those treated with prednisolone in combination with CST (early and late initiation). Although it was a higher mortality in the prednisolone alone group, this was of no significance (*p* = 0.14). This might reflect the fact that only 12 patients were treated with prednisolone alone, and it was impossible to obtain statistical significance. On the other hand, this again reflects that we adhered to our local treatment guidelines for the initiation of CST in BP patients.

The time of admission was shorter in this study (mean [± SD]: 8 [± 5] days) than in the study by Kibsgaard et al. (mean [± SD]: 14 [± 9] days) (**Table 2**). This reduction in admission time may indicate more effective management of the acute phase of the disease. Furthermore, we found a decreased initial dose of prednisolone (from a mean ± SD of 44 ± 15 mg in Kibsgaard et al.’s study to 25 ± 11 mg in this study). However, we did not find any changes in the overall remission or relapse (34% in Kibsgaard et al.’s study, 38% in this study) of BP between the two study periods.

The 1-year mortality of 17.2% in this study was similar to that of 16.7% reported in a study from Finland conducted by Försti et al. (7). Finland is comparable with Denmark in several aspects, particularly demographics (for instance, having an older age structure), the incidence of BP, and treatment methods for BP. The 1-year mortality for citizens aged >70 years was approximately 5.4% in 2018 in Denmark^2^. Thus, BP patients have an increased mortality rate compared with the general population of the same age group, which has been confirmed in several studies, including this study (14–16).

Considering the epidemiological aspects of this study, there was a decreased rate of CVDs of 46% versus 70% in Kibsgaard et al.’s study (10). Few studies have demonstrated an association between CVD and BP. However, Kibsgaard et al.’s study and Försti et al.’s of 198 BP patients (7) both found CVD as the most common comorbidity. This was also observed in the present study. Recent studies have demonstrated BP association with hypertension (17, 18). Lee et al. found a significant adjusted odds ratio of 2.03 [95% CI 1.24–3.32] of BP when having hypertension (18). The present study did not differentiate between different types of CVD; therefore, the proportion of patients with hypertension was unknown. However, the possible association between specific forms of CVD and BP is an interesting aim for further research.

In this study, CVD was the most common comorbidity, followed by type II DM (29%), cancer (25%), and neurological diseases (21%). There are hypotheses of cross-reactions against BP230 isotopes, where the autoantibody binds to both antigens expressed in neurological tissues and the basement membrane of human skin (19). This suggests an association between BP and neurodegenerative disorders. A larger case–control study from Denmark revealed a significantly increased frequency of multiple sclerosis (MS) among BP patients, with a risk of developing MS more than five times higher than that in the background population (19). As this was a retrospective descriptive study and not a follow-up study, we could not conclude any association between MS and BP based on our findings. Three patients of our cohort had MS as a comorbidity at the time of BP diagnosis.

As mentioned earlier, several drugs have been reported to increase the risk of developing BP, including antihypertensive drugs, anti-inflammatory drugs, diuretics, antirheumatic drugs, vaccines, and gliptins. The most frequent drugs used in our cohort were paracetamol (41%), acetylsalicylic acid (23%), and ACE inhibitors (21%). This may be explained by the advanced age and multiple comorbidities of our patients. In our cohort, 12% of the patients used gliptins, in total 18 received vildagliptin, one received linagliptin, and one received sitagliptin. In the last years of BP research, the use of dipeptidyl peptidase-4 inhibitors (particularly vildagliptin and linagliptin) for the treatment of DM has been investigated and has shown to be significantly associated with BP (20). The Danish Endocrine Society suggests alogliptin, linagliptin, and sitagliptin as first-line drugs if there is indication for using gliptins in the treatment of DM. Therefore, it is interesting that 18 out of 20 of our gliptin users were prescribed vildagliptin. Vildagliptin has in several studies been shown to have the highest association with BP compared with other gliptins (21). Lastly, there has been an increase in the use of non-insulin, glucose-lowering drugs such as gliptins the last decade in Denmark, and in 2021, 7.5% of the type II DM population in Denmark used gliptins (22). In comparison, we found 42% of our type II DM BP patients using gliptins. In conclusion, our results may support the rising evidence of gliptin-associated BP.

The strengths of this study were a long study period and the substantial percentage of patients (almost 90%) with a BP diagnosis based on histology, direct IF, and clinical presentation. The limitations of this study were the lack of assessment of BP severity, for example, using the ABSIS or BPDAI disease scores, which could have provided more specific information on the disease stage, and hence, the possible effect of early initiation of CST. Therefore, the results must be interpreted with caution. Furthermore, we could have confirmed the BP diagnosis by performing serological tests for autoantibodies against BP180 and/or BP230. Consequently, we have now adapted this as a routine practice in our department. Another limitation that may have affected our results was selection bias, as our department consulted the most severe cases within a limited geographic region. However, the fact that the same standard guidelines were used and that, in this study, these guidelines were followed quite consequently by the department specialists indicates the strength of this study and its results.

## 5 Conclusion

The rate of complications and 1-year mortality did not differ significantly between the groups that initiated early (<28 weeks) or late (>28 weeks) CST. However, the rate of relapse was lower in the group with early initiation of CST. When comparing the outcomes of patients treated with prednisolone alone with those of patients treated with CST, we found no difference.

Furthermore, we found that the initial dose of prednisolone and admission time were reduced in this study compared with those reported in a similar study conducted before the implementation of a local treatment guideline recommending the early initiation of CST. Thus, the initial prednisolone dose was reduced after a change in the treatment guidelines for BP patients; however, further studies are needed to clarify whether the reduced prednisolone dose lowers the risk of adverse outcomes and mortality in these patients.

## Data availability statement

The raw data supporting the conclusions of this article will be made available by the authors, without undue reservation.

## Ethics statement

Ethical review and approval was not required for the study on human participants in accordance with the local legislation and institutional requirements. Written informed consent for participation was not required for this study in accordance with the national legislation and the institutional requirements. Written informed consent was obtained from the individual(s) for the publication of any potentially identifiable images or data included in this article.

## Author contributions

RB, AF, CV conceived of the study, made the design, and coordinated the study. AF provided all patient data. GO collected the epidemiological data. GO and IF performed the interpretation of data. IF performed the statistical analysis, interpreted the results, and composed the discussion. RB, AF, CV contributed to drafting and critical revision of the manuscript. All authors contributed to the article and approved the submitted version.

## Footnotes

1 Lægehåndbogen at Sundhed.dk. Pemfigoid (2022). https://www.sundhed.dk/sundhedsfaglig/laegehaandbogen/hud/tilstande-og-sygdomme/bulloese-tilstande/pemfigoid/ [Accessed May 1, 2022].

2 «Number of deaths» from Statistic Denmark. (2018). https://www.dst.dk/en/Statistik/emner/borgere/befolkning/doedsfald [Accessed February 23, 2023].
